# ARE UNDERSERVED GROUPS INCLUDED IN AGING RESEARCH? RESULTS ACROSS MANY CLINICAL RESEARCH STUDIES

**DOI:** 10.1093/geroni/igae098.0407

**Published:** 2024-12-31

**Authors:** Rebecca Neiberg, Xiaoyan Leng, Denise Houston, Michael Miller, Kristen Beavers, W Jack Rejeski, Stephen Kritchevsky, Barbara Nicklas

**Affiliations:** Wake Forest University School of Medicine, Winston-Salem, North Carolina, United States; Wake Forest University School of Medicine, Winston-Salem, North Carolina, United States; Wake Forest University School of Medicine, Winston Salem, North Carolina, United States; Wake Forest University School of Medicine, Winston-Salem, North Carolina, United States; Wake Forest University School of Medicine, Winston-Salem, North Carolina, United States; Wake Forest University, Winston-Salem, North Carolina, United States; Wake Forest University School of Medicine, Wake Forest University School of Medicine, North Carolina, United States; Wake Forest University School of Medicine, Winston-Salem, North Carolina, United States

## Abstract

Research can be inherently biased when results cannot be generalized across different subsets of a population that differ from those enrolled in the study. Thus, it is critical to recruit a diverse range of individuals who represent the entire population and who represent the disease or health condition being studied. The purpose of this study was to examine the demographics of 3,454 individuals enrolled in 11 clinical studies conducted from 2002-2023 and supported by the Wake Forest Older Americans Independence Center. Studies included recruited >80 participants and had a minimum age eligibility of 55 years with no upper age exclusion. The total number of individuals phone screened for participation was 19,937. The characteristics of those enrolled were compared to those who were initially screened for participation to assess whether eligibility criteria enabled participants to approximately match the characteristics of those who inquired about participation. The demographics of the screened participants were: Age (mean±SD), 69.8±7.0 yrs; 69% female; 76.4% non-Hispanic white/23.6% black; and 79.3% with at least some college education. The enrolled participants were: Age, 69.8±6.6 yrs; 64% female; 76.1% non-Hispanic white/23.9% black; and 80.5% with some college education. The age distribution of those screened ranged from 23-98 years with 43.7% being >70 yrs, 19.9%> 75 yrs, and 6.8%> 80 yrs while the distribution of those enrolled ranged from 55-92 years with 45.2% being >70 yrs, 20.7%> 75 yrs, and 7.6%> 80 yrs. Further analyses will compare these demographics to those of the 5 counties surrounding the research center.

